# High doses of zoledronic acid induce differential effects on femur and jawbone microstructure

**DOI:** 10.1002/cre2.643

**Published:** 2022-08-07

**Authors:** Mariana Q. S. Soares, Jeroen Van Dessel, Reinhilde Jacobs, Gustavo Z. Ferreira, Paulo Sérgio da Silva Santos, Laura F. P. Nicolielo, Marco A. H. Duarte, Izabel R. F. Rubira‐Bullen

**Affiliations:** ^1^ Department of Surgery, Stomatology, Pathology and Radiology, Bauru School of Dentistry University of São Paulo Bauru Brazil; ^2^ OMFS‐IMPATH Research Group, Department of Imaging and Pathology Faculty of Medicine, KU Leuven and Oral and Maxillofacial Surgery, University Hospitals Leuven Leuven Belgium; ^3^ Division of Oral Radiology Faculdade São Leopoldo Mandic, Instituto de Pesquisa São Leopoldo Mandic Campinas Brazil; ^4^ Department of Dental Medicine Karolinska Institutet Stockholm Sweden; ^5^ Department of Surgery and Stomatology, School of Dentistry University Center of Maringá Maringá Brazil; ^6^ Department of Dentistry, Endodontics and Dental Materials, Bauru School of Dentistry University of São Paulo Bauru Brazil

**Keywords:** bisphosphonates, bisphosphonate‐associated osteonecrosis of the jaw, bone morphometry, micro‐computed tomography

## Abstract

**Objectives:**

The aim of this study is to investigate the long‐term effects on jaw and femur bone induced by oncologic doses of zoledronic acid in a young rat model.

**Material and Methods:**

Six 12‐week‐old male Wistar rats received zoledronic acid (0.6 mg/kg) and six control rats received saline solution in the same volume. Compounds were administered intraperitoneally in five doses every 28 days. Euthanasia was performed 150 days after therapy onset. After animal sacrifice, their mandibles and femurs were scanned ex vivo using a high‐resolution (14 μm) micro‐computed tomography. Morphometric bone parameters were calculated using CT‐Analyzer (Bruker, Belgium) between the first and second mandibular molars and in the distal femur metaphysis and epiphysis.

**Results:**

The treatment group as compared to the controls showed a significantly (*p* < .05) increased bone quantity (↑BV/TV, ↓Po[Tot], ↑Tb.Th), bone density (↑TMD, ↑BMD), and osteosclerosis of the trabecular bone (↓Tb.Sp, ↓Conn.Dn, ↓Tb.Pf, ↓SMI) in all anatomical sites. Bone remodeling suppression due to zoledronic acid treatment was more pronounced (*p* < .05) in the femoral metaphysis relative to the mandible and epiphysis. The exploratory linear discriminant analysis showed that for the mandible, it was mainly the bone quantity‐related morphometric indices (BV/TV and Tb.Th), while for the femoral epiphysis and metaphysis, it was bone structure‐related (Tb.Pf and Tb.N), which are of primary importance to study the treatment effect.

**Conclusion:**

High doses of bisphosphonates can differently affect the bone quantity, density, and structure in long bones and jawbones. In the metaphysis, bone changes were primarily concentrated in the region of the growth plate. Future studies may consider the use of bone morphometric indices to evaluate the effect of bisphosphonates.

## 1 INTRODUCTION

Bisphosphonates are potent inhibitors of bone remodeling, interfering with recruitment, differentiation, resorptive activity, and inducing apoptosis of osteoclasts (Russell, 2011). Intravenous nitrogen‐containing bisphosphonates have been successfully used in the treatment of bone metabolic diseases and in the prevention of skeletal‐related events (i.e., pathological fractures and bone pain) in oncologic patients with metastasis. These patients may remain on bisphosphonate treatment for extended periods (Lockwood et al., 2019). In children and adolescents, bisphosphonates are used in the treatment of osteogenesis imperfecta (Malmgren et al., 2020) and a variety of diseases that result in bone density decrease and malignancies (Cheung & Borno, 2020).

Uncommon late site‐specific side effects, such as atypical fracture of the femur and osteonecrosis of the jaw, have been related to long‐term therapies with high‐dose bisphosphonates in adults (Ruggiero et al., 2014). Although their pathogenesis remains largely unknown, preclinical and clinical investigations lead to several proposed mechanisms, including remodeling oversuppression (Cheung & Borno, 2020; Ruggiero et al., 2014), microdamage accumulation (Cho et al., 2018; Hoefert et al., 2010; Lockwood et al., 2019), decreased bone vascularization (Soares et al., 2018), and impaired local trauma repair (Jabbour et al., 2014; Shane et al., 2010).

Bisphosphonate treatments have also been associated with atypical fracture of the femur in children and adolescents (Boyce et al., 2017; Vasanwala et al., 2016), although controversy persists over the incidence in a younger population (Nasomyont et al., 2019; Vasanwala et al., 2016; Vuorimies et al., 2017). To date, no case of bisphosphonate‐related osteonecrosis of the jaw in children or adolescents has been reported in the literature (Hegazy et al., 2020). However, pediatric patients under bisphosphonate treatment are not free from jawbone remodeling suppression side effects, such as tooth eruption delay (Kamoun‐Goldrat et al., 2008; Malmgren et al., 2020). Young animal models have shown a relationship between bisphosphonate therapy and ankylosis and tooth eruption delay (Bradaschia‐Correa et al., 2007) in a dose‐dependent manner (Hiraga et al., 2010). Additionally, the report of a bisphosphonate‐related osteonecrosis of the jaw in a 19‐year‐old patient after tooth extraction associated with denosumab use, another bone‐modifying agent, raises the concern that young patients are not immune to this complication (Uday et al., 2018).

To investigate these bone site‐specific effects of bisphosphonates in a younger population, it is essential to obtain more information on bone remodeling in different anatomical bone sites. Micro‐computed tomography (CT) is a validated method for the high‐resolution evaluation of tridimensional bone structure, presenting high accuracy compared to histomorphometry (Soares et al., 2018). The impact of bisphosphonates on bone quality of adult animal models either with or without induced osteonecrosis has been largely investigated (Hatori et al., 2015; Zhang et al., 2019). Nevertheless, information on young animal models is limited and most research focuses on bisphosphonate's effect on bone growth (Battaglia et al., 2011; Rao et al., 2008). We hypothesize that high‐dose long‐term bisphosphonate administration may induce site‐specific microarchitectural changes during bone growth. The aim of this study is to investigate whether zoledronic acid differentially affects different bone morphology depending on the anatomical site. Second, an exploratory analysis was performed to indicate which parameters are best suited to detect changes in bone morphology.

## 2 METHODOLOGY

### 2.1 Animals and experimental design

Twelve‐week‐old healthy male Wistar rats weighing on average 343± 26 g were used. The animals were sterile shaved, housed individually under 12:12 light/dark cycles, at controlled temperature and humidity, and were allowed ad libitum access to solid food and water.

All animal procedures were performed in accordance with the guidelines of the ethics committee of the Bauru School of Dentistry of the University of São Paulo, Brazil (reference number 022/2014) and all data are reported according to the ARRIVE (Animal Research: Reporting of In Vivo Experiments) criteria (Percie du Sert et al., 2020).

Animals were randomly divided into two groups: a control and a zoledronic acid treatment group. The medication regimen was performed according to the recommendations of Maahs et al. (2011) and was considered equivalent to the human dose given to oncologic patients adjusted for rats' weight, metabolic rates, and treatment period (Pozzi et al., 2009). Rats in the treatment group were given a high dose of 0.6 mg/kg zoledronic acid every 28 days intraperitoneally (Zometa®, Novartis Pharma, Basel, Switzerland) for a total of five doses, whereas control animals received an equivalent volume of saline solution. Determination of body weight was performed before each injection to recalculate the exact solution volume. At 150 days, animals were anesthetized and euthanized with a combination of ketamine (Dopalen®; Vetbrands, Paulinia, Brazil) and xylazine hydrochloride (Anasedan®; Vetbrands). The right mandibles and femurs were disjointed, stripped of musculature, and immediately prepared for micro‐CT scanning.

### 2.2 Micro‐CT scanning

The hemimandibles were cut at the distal end of the third molar and mesial end of the first molars to fit in the field of view of the high‐resolution (14 µm^3^) scan protocol. Each sample was placed in a 1.5 ml Eppendorf tube with saline solution and scanned with a SkyScan1174 micro‐CT (Bruker, Kontich, Belgium). Scanning parameters were set at 50 kVp, 800 μA, frame averaging of 6° and 180° rotation with an angular step of 0.8°. A 0.5‐mm‐thick aluminum filter was used to reduce noise artifacts and to minimize beam hardening effects that could affect the further analysis. Hydroxyapatite phantoms of 0.25 and 0.75 g/cm^3^ (Bruker) were used and scanned according to the scan protocol to perform a BMD calibration with respect to the attenuation values. The cross‐sectional images were reconstructed from the projection images in NRecon (Bruker). After reconstruction, micro‐CT images were registered using a MeVisLab framework (MeVis Medical Solutions AG, Bremen, Germany) to spatially align all scans to ensure a uniform comparison of anatomical structures (Van Dessel et al., 2013).

### 2.3 Image analysis

**Figure 1:**
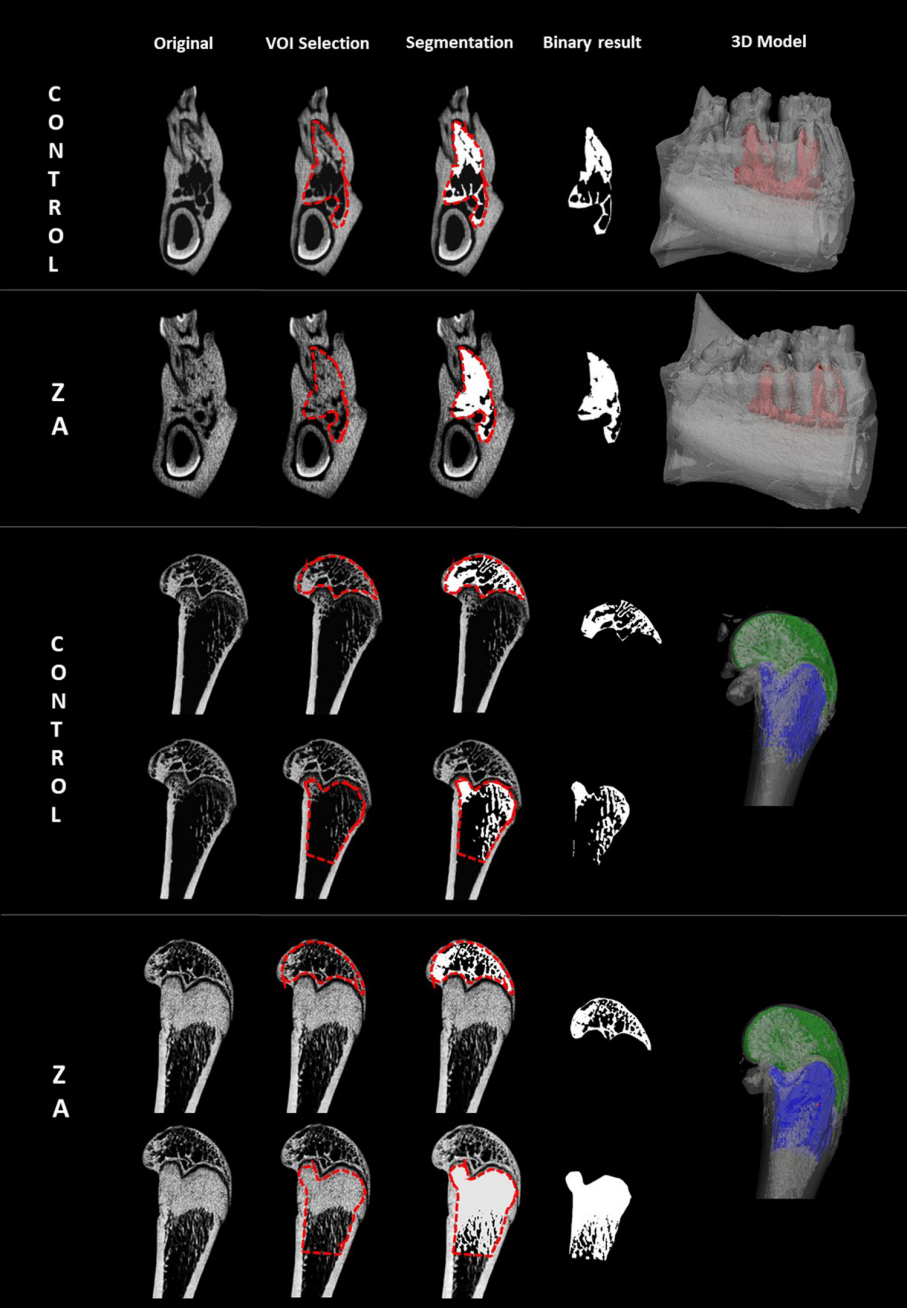
Image processing steps on micro‐computed tomography (CT) scans of zoledronic acid‐treated and control rat mandibles (upper row) and femur epiphysis and metaphysis (lower row). All micro‐CT images were spatially aligned in the same coordinate system. One mean volume of interest (red dashed line) comprising only trabecular bone was generated and used in all samples for morphometric bone analysis. Trabecular bone structures were automatically segmented and corresponding three‐dimensional models were rendered.

Guidelines of the American Society of Bone and Mineral Metabolism for assessment of bone microstructure in rodents using micro‐CT were taken into account during image and trabecular bone analysis (Bouxsein et al., 2010). All samples underwent the same image processing workflow shown in Figure 1 (Van Dessel et al., 2017). To guarantee a uniform comparison of trabecular bone structures between groups, it was decided to use a standardized volume of interest (VOI) for the mandible, distal epiphysis, and metaphysis of the femur. One general VOI was generated for the mandible consisting only of alveolar bone between the mesial and distal roots of the molars, and trabecular bone below the apex of the molars in all samples. Nearby anatomical structures such as the mandibular canal, cortical bone, periodontal ligament, and incisor tooth were excluded. In the distal femur, two VOIs were selected comprising only trabecular bone in (1) epiphysis and (2) metaphysis. Trabecular bone structures were segmented using an automatic adaptive mean threshold algorithm in CT‐Analyzer (Bruker). Computer‐suggested bone thresholds were visually reassessed after overlapping the segmented bone network on the original bone structures to confirm an accurate segmentation. From the resulting binary images, individual three‐dimensional (3D) models of the trabecular and cortical bone in the mandible and femur were made using CTVol (Bruker).

### 2.4 Morphometric bone parameter calculation

Morphometric bone indices were operator‐independent calculated and blinded for treatment regimen, based on the segmented trabecular bone structure and were grouped according to terms clinically used for bone quality evaluation (Van Dessel et al., 2016): (1) *Bone quantity*: bone volume fraction (BV/TV in %), total porosity percentage (Po[tot] in%), specific bone surface (BS/TV in %), and trabecular thickness (Tb.Th in mm); (2) *bone structure*: trabecular number (Tb.N in 1/mm), trabecular separation (Tb.Sp in mm), connectivity density (Conn.Dn in 1/mm^3^), degree of anisotropy (DA), trabecular pattern factor (Tb.Pf in 1/mm), and structure model index (SMI) as well as (3) *bone density*: tissue mineral density (TMD in mg HA/cm^3^) and bone mineral density (BMD in mg HA/cm^3^).

### 2.5 Statistical analysis

The minimum required sample size was calculated using the effect size of a previous comparison study between control and zoledronic acid‐treated rats with a similar design (Imada et al., 2018). A power analysis in G*Power 3.1 suggested a minimum sample size of 12 animals for a repeated‐measures multivariate analysis of variance with six groups (two treatments and three anatomical sites) and 10 morphometric parameters when assuming 90% power and *α* of .05 significance. A repeated‐measures multivariate analysis of variance was used to compare the morphometric indices between bone site (mandible, metaphysis, epiphysis) and group (zoledronic acid, control). Post hoc Bonferroni‐corrected tests were used to explore significant interaction effects. Mean and standard deviations of each parameter were reported. An exploratory linear discriminant analysis for each anatomical site was performed to identify the morphometric parameters that best predict group membership. The strength of the canonical correlation (cc) was used to indicate the discriminatory power of each morphometric parameter for group classification. Leave‐one‐out cross‐validation was used to assess model prediction performance and how the results of the LDA will generalize to an independent data set. Statistical analyses were performed in SPSS (version 22; IBM, New York, USA) at a significant level of .05.

## 3 RESULTS

### 3.1 Body weight and animals' health

All animals tolerated well the experiment with the absence of relevant adverse effects. No significant differences in body weight were found during treatment between groups. No clinical or radiographic signs of bisphosphonate‐related osteonecrosis of the jaw or atypical fracture of the femur were observed.

### 3.2 Bone morphometric analysis

#### 3.2.1 Treatment effect

Administration of zoledronic acid caused a significant overall change in bone quality in all bones (*F* = 59.8; *p* < .001 *η* ^2^_*p*_ = 0.98). Zoledronic acid‐treated rats showed an overall larger bone quantity (higher BV/TV and smaller Po[tot]) related to thicker (larger Tb.Th) and less complex trabeculae (smaller BS/TV). The trabecular bone structure after zoledronic acid treatment showed typical osteosclerotic characteristics marked by a smaller number of trabeculae (smaller Tb.N and Conn.Dn) and more plate‐like structure (lower SMI), explained by smaller trabecular spaces (smaller Tb.Sp), more enclosed cavities (smaller Tb.Pf), and heterogeneous density distribution (smaller DA). No significant changes in BMD were observed.

#### 3.2.2 Anatomical site effect

**Figure 2:**
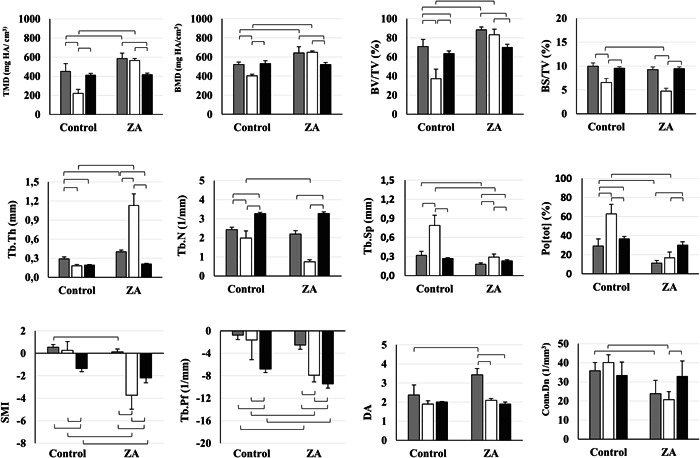
Overview of three‐dimensional morphometric bone parameters for zoledronic acid (ZA) and control groups in the mandible (gray), and cartilage in the metaphysis (white) and epiphysis (black) of the femur. ZA‐treated rats showed a more pronounced significant decrease in bone remodeling in the metaphyseal cartilage relative to the alveolar jawbone and epiphyseal cartilage. Bars display means and standard deviations. Lines indicate significant differences between groups. Zoledronic acid‐induced bone quantity increase with a more corticalized structure in both femur and mandible.

Without any influence of treatment, the mandible had a significantly denser bone quantity, structure, and density (*F* = 82.5; *p* < .001 *η* ^2^_*p*_ = 0.97) in comparison to femoral metaphysis and epiphysis (Figure 2).

#### 3.2.3 Interaction between factors

Bone remodeling suppression due to zoledronic acid treatment was more pronounced in the femur metaphysis relative to the jaw and femur epiphysis (Figure 2 and Table 1). Overall in the zoledronic acid group, the metaphysis presented a higher increase in bone quantity (an increase of 124% on BV/TV and 527% on Tb.Th) in relation to the mandible. No significant changes were observed in the epiphysis. A denser structure with a smaller number of trabeculae (decrease of 62% on Tb.N), smaller marrow spaces (decrease of 63% on Tb.Sp and 73% on Po[tot]) and a more connected structure (decrease of 391% on Tb.Pf) was also observed in the femur metaphysis in the zoledronic acid group. In the mandible, a significantly smaller number of marrow spaces (decrease of 73% on Po[tot]) and higher DA were observed (an increase of 44% on DA). No significant change was observed in the epiphysis.

**Table 1:** Mean and standard deviations of quantity‐, structure‐ and density‐related morphometric parameters for the three anatomical locations

		Mandible			Metaphysis			Epiphysis		
Morphometric parameter	Unit	Control	ZA	*p* Value	Control	ZA	*p* Value	Control	ZA	*p* Value
Bone quantity related										
Bone volume fraction	(%)	70.8 ± 7.5	88.4 ± 2.9	**<.001**	37.1 ± 10.1	83.1 ± 6.0	**<.001**	63.5 ± 2.7	69.9 ± 3.4	.28
Total porosity percentage	(%)	29.2 ± 7.5	11.6 ± 2.9	**<.001**	62.9 ± 10.1	16.9 ± 6.0	**<.001**	36.5 ± 2.7	30.1 ± 3.4	.28
Specific bone surface	(%)	9.9 ± 0.7	9.24 ± 0.5	.79	6.6 ± 0.9	4.7 ± 0.6	**<.01**	9.5 ± 0.3	9.4 ± 0.4	.66
Trabecular thickness	(mm)	0.29 ± 0.03	0.40 ± 0.03	**<.001**	0.18 ± 0.02	1.1 ± 0.2	**<.001**	0.19 ± 0.01	0.21 ± 0.01	.76
Bone structure related										
Trabecular number	(1/mm)	2.4 ± 0.1	2.2 ± 0.2	.45	2.0 ± 0.4	0.7 ± 0.1	**<.001**	3.3 ± 0.1	3.27 ± 0.1	.83
Trabecular separation	(mm)	0.32 ± 0.06	0.18 ± 0.02	**<.001**	0.79 ± 0.16	0.29 ± 0.05	**<.001**	0.27 ± 0.01	0.23 ± 0.02	.11
Connectivity density	(1/mm^3^)	35.8 ± 4.2	23.8 ± 7.0	**<.05**	40.1 ± 4.1	20.7 ± 4.2	**<.001**	33.3 ± 7.0	32.9 ± 8.1	.92
Degree of anisotropy		2.4 ± 0.5	3.4 ± 0.3	**<.001**	1.9 ± 0.2	2.1 ± 0.1	.32	2.0 ± 0.03	1.9 ± 0.1	.89
Trabecular pattern factor	(1/mm)	−0.76 ± 0.78	−2.52 ± 0.77	**<.01**	−1.61 ± 3.55	−7.92 ± 1.19	**<.01**	−6.82 ± 0.60	−9.43 ± 0.78	**<.001**
Structural model index		0.54 ± 0.23	0.12 ± 0.26	**<.05**	0.26 ± 0.79	−3.73 ± 1.25	**<.001**	1.36 ± 0.27	−2.20 ± 0.42	**<.01**
Bone density related										
Tissue mineral density	(mg HA/cm^3^)	450.0 ± 82.5	585.5 ± 56.6	**<.001**	221.4 ± 40.9	564.0 ± 29.7	**<.001**	410.8 ± 19.8	415.2 ± 16.9	.94
Bone mineral density	(mg HA/cm^3^)	522.8 ± 23.3	643.3 ± 64.6	**<.001**	403.0 ± 15.9	650.0 ± 14.2	**<.001**	530.8 ± 29.6	520.5 ± 21.6	.68

*Note*: Bold values are statistically significance either <.05, <.01 or <.001.

Abbreviation: ZA, zoledronic acid.

In agreement with these findings increase in TMD and BMD was observed in the zoledronic acid group the metaphysis (an increase of 154% and 61%) and in the mandible (an increase of 30% and 23%) compared to the control group.

#### 3.2.4 Group classification based on morphometric parameters

All bone specimens of the three anatomical sites were correctly classified in the corresponding treatment group. Cross‐validation showed a 100% prediction accuracy for the metaphysis, 83.3% for the mandible and 72.7 for the epiphysis. The distinctive importance of the morphometric parameters was specific for each anatomical site. For the mandible, BV/TV (cc = 0.88), Tb.Th (cc = 0.80), Tb.Sp (cc = −0.65), and DA (cc = 0.64) were more important for the performance of the given discriminant model, while for the metaphysis Tb.Pf (cc = −0.79), SMI (cc = −0.66), Tb.N (cc = 0.63) were the most important distinctive parameters. For the epiphysis, the cc parameters were smaller Tb.Pf (cc = 0.60), BV/TV (cc = 0.58), and Tb.N (cc = 0.57) in comparison to the metaphysis and mandible.

## 4 DISCUSSION

Over the past few years, bisphosphonate indications for the treatment of several chronic metabolic bone conditions have emerged, being increasingly used in young populations (Simm et al., 2018). Hence, it is important to understand the impact of bisphosphonate therapy on different bone sites and to identify objective parameters that could contribute to measuring those impacts. In this study, for the first time, three‐dimensional morphometric parameters were used to predict whether zoledronic acid differentially affects bone characteristics in three anatomical sites. A higher prediction accuracy was observed in the femur metaphysis and in the mandible. When evaluating the parameters individually, the methaphysis effects of zoledronic acid were mainly predicted by trabecular structure changes, while in the mandible, bone quantity parameters were most accurate. These results support the notion of bisphosphonate site‐specific effect on trabecular bone architecture.

Previous studies in adult patients have demonstrated that weight‐bearing sites, such as tibia, are more susceptive to bisphosphonate antiresorptive effect (Burghardt et al., 2010; Chapurlat et al., 2013). Zoledronic acid reduction of bone turnover markers has also been shown to be more prominent in the long bone than in the jaw (Vermeer et al., 2017). Apparently, the bone structure also varies within the bones. In the femur epiphysis, no significant change in bone architecture was observed. In the metaphysis, mineral deposition was concentrated in the region of the growth plate, also known to be the denser region in the long bones (Rao et al., 2008). The differential drug effect in the metaphysis would be expected in growing animals like the ones evaluated in this study considering that the femur undergoes endochondral bone formation (Vermeer et al., 2017). This process requires cartilage ossification and posterior replacement by bone tissue in the metaphysis, which is dependent on osteoclast activity (Vermeer et al., 2017). Nitrogen‐containing bisphosphonates have been shown to hamper the replacement of calcified cartilage leading to thicker and denser growth plates on radiographic images of younger animals (Vermeer et al., 2017). In contrast, the region of alveolar bone undergoes intramembranous ossification in a process of preosteogenic condensations of mesenchymal cell‐guided Meckel's cartilage (Rezende et al., 2017).

Preclinical studies have demonstrated that bisphosphonates have A high atypical fracture of the femur and induce higher remodeling suppression in the jaws compared to other bones (Wen et al., 2011). Our results showed that in the controls, the mandible also presents a denser structure compared to the femur metaphysis. These pre‐existing differences in bone structure density may also lead to site disparity in bisphosphonate absorption and concentration. Wen et al. (2011) demonstrated a higher bisphosphonate uptake and release in the mandible compared to appendicular (humerus, radius/ulnar, femur, tibia/fibula) and axial (ribs, vertebrae) bones in rats (Wen et al., 2011). A higher concentration of bisphosphonates in the jaw microenvironment could also lead to higher cytotoxicity to different cell types.

In our investigation overall, in the zoledronic acid group, the mandible also presented denser bone with smaller marrow spaces compared to femur metaphysis. This result is in accordance with Vermeer et al. (2017), who also reported a reduction in the marrow cells in the jaw, but not in the long bone (Vermeer et al., 2017). The markedly limited marrow spaces in the jaw may lead to a diminution in the number of marrow cells (Vermeer et al., 2017) and to a decrease in vascularity (Soares et al., 2018). This hypothesis should be further confirmed by histological analysis.

Evidence shows that lower doses of bisphosphonates improve bone volume fraction and increase the trabecular number, while higher doses increase the trabecular thickness (Gou et al., 2014). The constant deposition of mineral content may lead to the fusion of the trabecular structures. This process would be facilitated in denser regions where the trabecular separation is already smaller and would explain the substitution of the trabecular structure by a cortical structure in bones submitted to a bisphosphonate high‐dose protocol.

There are several bone disorders affecting young patients in which bisphosphonate treatment has been recommended, including osteogenesis imperfecta, idiopathic juvenile osteoporosis, secondary osteoporosis, fibrous dysplasia, and skeletal neoplasms (Simm et al., 2018). It is important to consider that these patients may undergo long‐term treatment. Even after discontinuing treatment, bisphosphonates may remain in the bone for several years (Gou et al., 2014). Zoledronic acid was administered at a dose similar to the oncologic doses in children (Simm et al., 2018), and in a long‐term scheme, corresponding to 10 human years (Sengupta, 2013). This protocol was also previously used to induce osteonecrosis of the jaw model in the presence of tooth extraction (Maahs et al., 2011). There are inherent challenges in the investigation of long‐term conditions in children related to the ethnic and physiological aspects and to the retention of the patients during the study (Kern, 2009). The human population is notoriously heterogeneous and is exposed to many factors that can contribute to the physiopathology of several diseases, making this an ever‐changing scenery as technology and research tools continue to advance the understanding of the basis of most diseases (Mcgonigle & Ruggeri, 2014). The results of the preclinical models are valuable for to investigate the effects of bisphosphonate on bone and related side effects. Although the inherent challenges from translating it to humans should be taken into account.

The present study showed that high‐dose, long‐term zoledronic acid treatment had a site‐specific effect on the mandible and femur during bone growth. Clinically, however, osteonecrosis only occurs in the jaw. The difference between long bones and jawbones, besides their mode of ossification (Simm et al., 2018) is the open environment of the oral cavity. Most likely, other factors such as oral diseases or angiogenesis inhibition play a more crucial role in the site‐specific nature of medication‐related osteonecrosis of the jaw (Vermeer et al., 2017).

This study has some limitations that need to be considered when interpreting its findings. First, the analysis was limited to ex vivo micro‐CT imaging. This meant that the process of bone remodeling throughout rat development could not be studied. Longitudinal dynamic bone morphometry may give more insight into bone remodeling changes due to bisphosphonate treatment. Second, our experimental model received one bisphosphonate dose scheme. Using different drug doses and intervals may add information to the discussion of the optimal bisphosphonate regimen considering its risks and benefits. Third, despite the different bone composition between the mandibula and the maxilla, the bone morphometric parameters of the maxilla were not analyzed in this study. Due to the fact that there are too many additional differences between the maxilla and the studied bone types that not be corrected for in the present study. Previous research has shown that the bone turnover in the mandible is similar to that in the femur and significantly lower than that in the mandible (Ristow et al., 2014). Moreover, it is primarily the mandibula that, like the femur, is the stress‐bearing anatomy that must withstand loading and microdamage that may result from the administration of bisphosphonates or other antiresorptive agents (George et al., 2018). Lastly, due to the limited statistical power, the results of the linear discriminant analysis are exploratory and should be interpreted with caution. Nevertheless, they strengthen the bone site‐ and treatment‐specific findings

In conclusion, we demonstrated differential site‐specific effects of zoledronic acid in the long bones and jawbones during bone growth in a rat model. In the femur distal metaphysis, the changes are primarily concentrated in the region of the growth plate. Future studies may consider the use of bone morphometric indices to evaluate the effect of bisphosphonates.

## AUTHOR CONTRIBUTIONS

All authors contributed to the study's conception and design. Material preparation, data collection, and analysis were performed by Mariana Quirino Silveira Soares, Jeroen Van Dessel, Gustavo Zanna Ferreira, Paulo Sérgio da Silva Santos, Laura Ferreira Pinheiro Nicolielo, and Marco Antônio Húngaro Duarte. The first draft of the manuscript was written by Mariana Quirino Silveira Soares and all authors commented on previous versions of the manuscript. This research was supervised by Reinhilde Jacobs and Izabel Regina Fischer Rubira‐Bullen. All authors read and approved the final manuscript.

## CONFLICT OF INTEREST

The authors declare no conflict of interest.

## Data Availability

The data that support the findings of this study are available on request from the corresponding author.
